# Pilot-Scale Production of Sericin-Derived Oligopeptides (SDOs) from Yellow Silk Cocoons: Peptide Characterization and Specifications

**DOI:** 10.3390/foods14030500

**Published:** 2025-02-05

**Authors:** Papungkorn Sangsawad, Surangkhanang Chumee, Phanthipha Laosam, Sittiruk Roytrakul, Sasikan Katemala, Manote Sutheerawattananonda

**Affiliations:** 1 School of Animal Technology and Innovation, Institute of Agricultural Technology, Suranaree University of Technology, Nakhon Ratchasima 30000, Thailand; 2 Postharvest Technology and Innovation in Animal Unit, Institute of Agricultural Technology, Suranaree University of Technology, Nakhon Ratchasima 30000, Thailand; laos.phanthipha@gmail.com; 3 School of Food Technology, Institute of Agricultural Technology, Suranaree University of Technology, Nakhon Ratchasima 30000, Thailand; palm_chumee@hotmail.com; 4 Research and Development Institute, Suranaree University of Technology, Nakhon Ratchasima 30000, Thailand; 5 Functional Proteomics Technology Laboratory, National Center for Genetic Engineering and Biotechnology, National Science and Technology Development Agency, Pathumthani 12120, Thailand; sittiruk@biotec.or.th; 6 Faculty of Agriculture at Kamphaeng Saen, Kasetsart University, Kamphaeng Saen Campus, Nakhon Pathom 73140, Thailand; sasikan.kat@ku.ac.th

**Keywords:** sericin-derived oligopeptides (SDOs), up-scaling, ACE inhibitor, DPP-IV inhibitory, sericin active peptides, yellow silk protein, insect protein, sericin

## Abstract

Our previous research demonstrated the health benefits of sericin-derived oligopeptides (SDOs) from yellow silk cocoons, particularly their hypoglycemic and antihypertensive properties. This study aims to produce SDOs at a pilot scale, preparing them for large commercial production as a novel food ingredient, and investigates the impact of scale-up on their characteristics and specifications. We compared the productivity of SDOs generated from 25 L and 300 L batches via the hydrolysis of sericin using 5% Neutrase (E/S) at 50 °C for 4 h. The 300 L production scale outperformed the 25 L scale, achieving a hydrolysis degree (DH) of 8.63%, a solid recovery rate of 94.35%, and enhanced inhibitory actions for dipeptidyl peptidase IV (DPP-IV) and angiotensin-converting enzyme (ACE). The characterization of peptides was carried out in ultrafiltered SDOs. Peptides < 3 kDa demonstrated optimal enzyme inhibition and were then fractionated by size exclusion chromatography into nine distinct fractions. Of the nine fractions, F1, F8, and F9 had significant enzyme inhibitory activity. LC-MS/MS analysis revealed 32 unique peptide sequences, with YPDLPYH exhibiting significant dual inhibitory effects on both DPP-IV (IC_50_ 1.35 mM) and ACE (IC_50_ 18.10 μM). The maximum residue limit (MRL) for trace metals, pesticide residues, and microbiological contamination in SDOs complies with food regulations. SDOs exhibited stability at 4, 25, and 45 °C for six months, based on their physical characteristics and biological activity. Considering their investigated characteristics, SDOs could be manufactured at a pilot capacity and used as a functional food component in commercial applications designed to improve metabolic health.

## 1. Introduction

The rising worldwide prevalence of metabolic disorders has increased the need for functional food ingredients that may alleviate chronic ailments, including type 2 diabetes mellitus (T2DM) and hypertension, via dietary intervention [1,2]. The presence of hypertension in T2DM people significantly elevates the risks of cardiovascular diseases (CVDs) [1,3]. Projections for 2045 estimate that an increase of 46% of the world population (783 million people) from 2021 will be affected by T2DM [4]. Between 2025 and 2050, CVD prevalence will increase by 90.0%, and gross mortality is expected to rise by 73.4%, resulting in about 35.6 million CVD deaths by 2050 [5]. The epidemiological data indicate that T2DM and CVDs may either be precursors or coexist. Studies on diabetes and hypertension have investigated and reported on two important enzymes: dipeptidyl peptidase IV (DPP-IV) and angiotensin-converting enzyme (ACE) [6,7,8]. DPP-IV is a metabolic enzyme present in the circulation. It degrades and inhibits incretins glucagon-like peptide-1 (GLP-1) and glucose-dependent insulinotropic polypeptide (GIP) [9]. This reduces insulin secretion postprandially [10]. Elevated DPP-IV activity adversely impacts T2DM patients, leading to hyperglycemia after carbohydrate consumption. The inactivation of DPP-IV enhances the availability of GLP-1 and GIP, which are crucial for the function of pancreatic beta cells to increase insulin production, leading to lowered blood sugar levels. DPP-IV inhibitory peptides, similar to GLP-1 agonists, demonstrate antidiabetic effects via the activation of the GLP-1 receptor. This stimulation results in increased insulin secretion, hence improving glycemic control in persons with T2DM. ACE plays a central role in blood pressure regulation and glucose homeostasis [2]. The development of hypertension is related to the conversion of angiotensin I into angiotensin II via the ACE system [11]. ACE inhibitors are commonly prescribed to manage these conditions; their prolonged use may result in various adverse effects, prompting the search for safer alternatives derived from natural sources and produced by food-grade enzymes [3,12].

Due to their natural origin, specific biological activities, and potential for incorporation into everyday food products, food-derived bioactive peptides have emerged as promising functional ingredients [13,14]. Among potential sources, sericin, an organic silk protein from yellow silk cocoons, traditionally considered a waste product [15], especially in the local silk textile industry in Thailand, presents an opportunity for sustainable food ingredient development. It could be converted into bioactive peptides, which not only adds value to this byproduct but also aligns with current trends in sustainable food production [16,17,18]. Their small size, specificity, selectivity, efficacy, and less systemic toxicity contribute to their preference over small-molecule drugs [19]. SDOs from yellow silk cocoons demonstrate several health benefits, especially hypoglycemic and antihypertensive properties, as demonstrated by our prior research. Initially, we established that SDOs possess dual inhibitory activity against both ACE and DPP-IV enzymes, with notable stability during simulated gastrointestinal digestion [20]. Additional research revealed that SDOs had significant hypoglycemic effects in streptozotocin (STZ)-induced diabetic rats [21]. They were also able to maintain blood pressure in a youthful and healthy range in aging rats during an oral chronic toxicity study, without adverse effects observed in histopathological examinations [22]. These promising results from laboratory-scale SDOs preparations call for further research into pilot-scale production, thorough peptide characterization, and SDO bioactivities during storage.

For more useful applications in manufacturing and quality control, we used liquid chromatography-tandem mass spectrometry (LC-MS/MS) to find and describe the specific peptide sequences that are responsible for their bioactivities and identity [23]. This lets us map out peptide sequences from bioactive fractions in great detail. Comprehending the structure-function relationship of these specific peptide sequences is essential for enhancing their preventative and therapeutic effectiveness, as well as for supplying crucial data for determining and characterizing of SDOs in manufacturing and quality assurance. The multifunctional characteristics of SDOs, demonstrating both ACE and DPP-IV inhibitory activities [20], offer a distinctive opportunity for the development of dual-action preventive and therapeutic agents, potentially improving homeostasis and leading to effective approaches to treatment for patients with concurrent hypertension and diabetes.

Following the successful laboratory-scale studies demonstrating SDOs’ hypotensive and hypoglycemic bioactivities in animal models, the transition to large-scale production presents several challenges that need to be addressed in order to evaluate their potential application to industry. In compliance with Thai FDA and international standards, the key challenge is maintaining the bioactivity and production of these peptides throughout scale-up operations, while also ensuring their shelf-life stability and safety assessments as novel food components. It is essential to comprehensively examine the effects of enhanced production capacity on the extent of hydrolysis, solid recovery, and inhibitory actions against DPP-IV and ACEs. Comprehending these aspects is essential for ensuring the consistency and efficacy of SDOs generated at industrial capacities. In the production of SDO powder, one of the crucial steps is spray drying. We need to determine the drying settings used and examine their impact on the physical characteristics of spray-dried SDO powder, including moisture content, water activity, and bioactivity. Safety assessments of SDOs in compliance with food standards and regulations highlight significant public health concerns, including total amino acid concentrations and permissible contaminant thresholds [24,25]. Additionally, as bioavailability frequently acts as a limiting factor in the efficacy of peptides administered orally, it is essential to address this fundamental challenge [26]. In silico models of gastrointestinal digestion can predict the stability and possible bioactivity of peptides after passage through the digestive system, enabling the determination of the effective dosage for newly developed products requiring additional processing [27]. Moreover, analyzing long-term stability profiles is crucial for determining appropriate storage conditions, logistics, and shelf life of SDO-based functional dietary supplements [28].

This study seeks to establish SDOs as functional food ingredients via pilot-scale production by investigating three essential facets of product characterization and quality control: (1) evaluating the effects of industrial-scale production on productivity for economic assessment, (2) identifying the bioactive peptides in SDO powder and their sequences to confirm product identity and compositional efficacy, and (3) validating food safety parameters in compliance with food standards, including stability under diverse storage temperatures to inform long-term storage, shipping, and distribution, thus ensuring safety and efficacy throughout the supply chain. This study thoroughly investigated essential topics to aid in the development of SDO-based supplements that might provide alternative, perhaps safer, and more effective solutions for addressing common metabolic illnesses.

## 2. Materials and Methods

### 2.1. Scale-Up Production and Process Optimization

#### 2.1.1. Pilot-Scale Production Process (25 L and 300 L)

**Figure 1:**
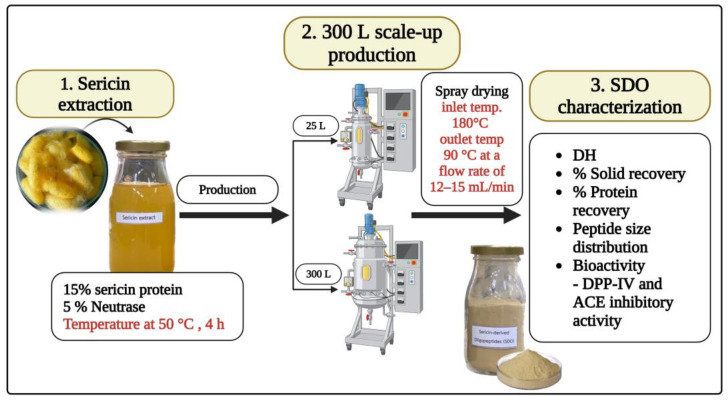
A schematic representation of the industrial scale-up process for producing bioactive peptides from sericin. The process consists of three main stages: (1) initial sericin extraction from silk cocoons resulting in a solution containing 15% sericin protein and 5% Neutrase, (2) up-scaling production using 25 L and 300 L reactors followed by spray drying, and (3) characterization of the final product using multiple parameters including degree of hydrolysis, solid recovery, protein recovery, peptide size distribution, and bioactivity measurements (DPP-IV and ACE inhibitory activities).

In our previous research, we established the SDO production process, including enzyme selection and optimal hydrolysis conditions. Figure 1 illustrates that enzyme types and the optimal conditions for SDO production have been established from our prior study [20,29]. A sericin protein concentration of 15% (*w*/*w*) and 5% Neutrase (E/S) relative to the sericin protein content was applied at a temperature of 50 °C for a period of 4 h. These conditions were then scaled up to 25 L and 300 L production capacities at the Fermentation Technology Research and Service Center, Faculty of Agro-Industry, Kasetsart University (Bang Khen, Bangkok, Thailand). Afterward, the samples were evaluated for solid and protein content (Kjeldahl method) by following Baur and Ensminger [30]. The degree of hydrolysis (DH), peptide size distribution (molecular weight distribution), and bioactivity, including DPP-IV and ACE activity, were also analyzed according to Sangsawad et al. [20].

The SDOs from the 25 L production scale were subjected to centrifugation at 10,000× *g* and then dried using a spray dryer (BUCHI model B-290, Flawil, Switzerland) with an inlet temperature of 180 °C and an exit temperature of 90 °C at a flow rate of 12–15 mL/min. The 300 L production scale-up SDOs underwent filtration with a cheese cloth and drying with a spray dryer (SPRAY DRYER SDG-100 NF) at 180 °C with an outlet temperature corresponding to the feed rate by Euro Best Technology Co., Ltd. (Pathumtani, Thailand). The subsequent analyses involved determining the moisture content by drying the samples at 105 °C until they reached a stable weight [30]. The inhibitory activity of DPP-IV and ACE were assessed by dissolving all protein hydrolysate powder samples in warm water at 90 °C, centrifuging at 10,000× *g* for 10 min, and then analyzing the supernatant for the enzyme activity.

The solid recovery and protein recovery were calculated as follows:

(1) $$\mathrm{Solid}\;\mathrm{recovery}\;\left(\%\right)=\frac{\mathrm{Solid}\;\mathrm{content}\;\mathrm{of}\;\mathrm{tratment}}{\mathrm{Total}\;\mathrm{solid}\;\mathrm{of}\;\mathrm{the}\;\mathrm{original}\;\mathrm{sample}\;}\times 100$$

(2) $$\mathrm{Protein}\;\mathrm{recovery}\;\left(\%\right)=\frac{\mathrm{Solid}\;\mathrm{content}\;\mathrm{of}\;\mathrm{tratment}}{\mathrm{Total}\;\mathrm{protein}\;\mathrm{of}\;\mathrm{the}\;\mathrm{original}\;\mathrm{sample}\;}\times 100$$

The fraction of peptide bonds in a protein that has been broken down is indicated by DH. The value was determined using the trinitrobenzenesulfonic acid (TNBS) method, as outlined by Boonkong et al. [31], which specifically detects α-amino groups. The calculation is based on quantifying these α-amino groups.

(3) $$\mathrm{Degree}\;\mathrm{of}\;\mathrm{hydrolysis}\;\left(\%\right)=\frac{\alpha -\mathrm{amino}\;\mathrm{acid}\;\left(4\;h\right)-\alpha -\mathrm{amino}\;\mathrm{acid}\;\left(0\;h\right)}{\mathrm{Total}\;\alpha -\mathrm{amino}\;\mathrm{acid}\;\left(\mathrm{original}\;\mathrm{sample}\right)\;}\times 100$$

#### 2.1.2. Bioactive Properties Analysis

##### ACE Inhibitory Activity Assay

The ACE inhibition assay following Laosam et al. [32] involves a reaction where Reagent A (ACE, 10 µL at 1 mU/mL) combines with Reagent B (test sample, 20 µL) and undergoes preincubation at 37 °C for 5 min. Subsequently, Reagent C (FAPGG substrate, 80 µL at 0.5 mM) was added to initiate the reaction. During the reaction, ACE hydrolyzes FAPGG, producing FAP and GG products, which causes a measurable decrease in absorbance at 340 nm. The reaction’s progression was observed for 30 min utilizing a Varioskan LUX microplate reader (Thermo Scientific, Vantaa, Finland). A positive control was prepared by substituting the test sample with deionized water. The reaction rate is expressed as ΔAbsorbance/min, from which the inhibitory activity was calculated using the following formula, and inhibitory concentration at 50% (IC_50_) was also determined:

(4) $$\mathrm{ACE}\;\mathrm{inhibitory}\;\mathrm{activity}\;\left(\%\right)=\frac{\mathrm{Slope}\;\left(\mathrm{positive}\;\mathrm{control}\right)-\mathrm{Slope}\;\left(\mathrm{test}\;\mathrm{sample}\right)}{\mathrm{Slope}\;\left(\mathrm{positive}\;\mathrm{control}\right)\;}\times 100$$

##### DPP-IV Inhibitory Activity Assay

The DPP-IV inhibition assay measures peptide inhibitory activity using a fluorescent GPN substrate system by following [33]. Reagent A (DPP-IV enzyme, 10 µL at 0.01 U/mL) was combined with Reagent B (test sample, 20 µL) and preincubated at 37 °C for 5 min in a 96-well microplate. Then, Reagent C (GPN substrate, 50 µL at 30 mM) was added to initiate the reaction. After 30 min of incubation at 37 °C, the mixture’s release of p-nitroanilide produced a fluorescent signal that could be detected at 405 nm using a Varioskan LUX microplate reader (Thermo Scientific, Vantaa, Finland). A blank control was prepared by replacing the test sample with deionized water. The DPP-IV inhibitory activity was calculated using the following formula:

(5) $$\mathrm{DPP}-\mathrm{IV}\;\mathrm{inhibitory}\;\mathrm{activity}\;\left(\%\right)=\frac{\mathrm{Slope}\;\left(\mathrm{positive}\;\mathrm{control}\right)-\mathrm{Slope}\;\left(\mathrm{test}\;\mathrm{sample}\right)}{\mathrm{Slope}\;\left(\mathrm{positive}\;\mathrm{control}\right)\;}\times 100$$

### 2.2. Product Characterization and Bioactivity Analysis

#### 2.2.1. Ultrafiltration (UF)-Based Separation

Using the ultrafiltration method test modified by Laosam et al. [32], SDOs from the production capacity expansion experiment were subjected to size separation. The Vivaspin 20 membrane with a molecular weight cut-off (MWCO) (Merck Millipore, Darmstadt, Germany) of 10 kDa was used, with 5 mL of the sample pipetted and centrifuged at 7000× *g* at 4 °C for 1 h. The retentate was then diluted with 5 mL of deionized water and centrifuged under the same conditions (This step was repeated twice). The permeate was then passed through a Vivaspin 20 membrane with a 3 kDa MWCO (Merck Millipore, Darmstadt, Germany) and centrifuged under the same conditions for 2 h. After that, 5 mL of deionized water was added and centrifuged under the same conditions (This step was repeated twice). This process enabled the separation of peptides > 10 kDa, between 3 and 10 kDa, and <3 kDa. Each sample was analyzed for α-amino acid content and inhibitory activity of DPP-IV and ACEs.

Using the following formula, the percentage of peptide yield obtained from ultrafiltration (UF) can be calculated:

(6) $$\mathrm{Peptide}\;\mathrm{recovery}\;\mathrm{of}\;\mathrm{UF}\;\mathrm{fraction}\;\left(\%\right)=\frac{\alpha -\mathrm{amino}\;\mathrm{of}\;\mathrm{fraction}}{\alpha -\mathrm{amino}\;\mathrm{of}\;\mathrm{the}\;\mathrm{initial}\;\left(\mathrm{crude}\right)\;\mathrm{sample}\;}\times 100$$

#### 2.2.2. Separation by Size Using Size Exclusion Chromatography (SEC)

The most potent ACE and DPP-IV inhibitory effects were demonstrated by the peptide fraction <3 kDa MWCO (UF3). Consequently, it was further fractionated by molecular size utilizing size exclusion chromatography, as adapted from Luasiri et al. [34] and Khongla et al. [35]. In order to achieve the separation, 100 µL of the material was injected through a Superdex Peptide 10/300 GL column (10 × 300 mm, GE Healthcare, Piscataway, NJ, USA) using a Fast Protein Liquid Chromatography (FPLC) system (AKTA explorer, GE Healthcare, Uppsala, Sweden). Deionized water was used as Solvent A for elution, whereas 30% acetonitrile (ACN) with 0.1% trifluoroacetic acid (TFA) at a flow rate of 0.8 mL/min was utilized as Solvent B. The elution conditions were implemented in a stepwise manner: 100% B for 3–3.75 mL, 2.5% B for 3.75–6 mL, 100% B for 6–6.75 mL, 2.5% B for 6.75–8 mL, and 100% B for 8–14 mL. Samples were collected, and the mobile phase was removed from each fraction using freeze-drying (Freeze Dryers, CHRIST/Gamma 2–16 LSC, Martin Christ Gefriertrocknungsanlagen GmbH, Osterode am Harz, Germany) prior to the analysis of the α-amino group content and ACE and DPP-IV inhibitory activities. The percentage of peptide recovery from SEC was calculated using the following formula:

(7) $$\mathrm{Peptide}\;\mathrm{recovery}\;\mathrm{of}\;\mathrm{SEC}\;\left(\%\right)=\frac{\alpha -\mathrm{amino}\;\mathrm{of}\;\mathrm{the}\;\mathrm{fraction}}{\mathrm{Initial}\;\alpha -\mathrm{amino}\;\mathrm{of}\;\mathrm{the}\;\mathrm{sample}\;(<3\;\mathrm{kDa})}\times 100$$

#### 2.2.3. Peptidomics Characterization of SDOs by Liquid Chromatography-Tandem Mass Spectrometry (LC-MS/MS)

Fractions 1, 8, and 9 (F1, F8, and F9), which included both soluble and insoluble peptides, showed notable inhibitory effects and were subjected to amino acid sequencing using LC-MS/MS in accordance with Laosam et al. [32]’s approach. The peptide samples were solubilized in 0.1% (*v*/*v*) formic acid and subsequently injected into an Ultimate 3000 capillary LC system (Dionex, Camberley, UK) coupled with an electrospray ionization (ESI)/quadrupole ion trap mass spectrometer (Model Amazon SL, Bruker, Germany) for peptide separation using a reverse-phase column (Hypersil GOLD 50 mm × 30.5 mm, 5 μm C18, Thermo Fisher Scientific, Waltham, MA, USA) and a guard column (Hypersil GOLD 30 mm × 30.5 mm, 5 μm C18, Thermo Fisher Scientific, Waltham, MA, USA) at a flow rate of 300 nL/min. The mass of the isolated peptides was examined using QToF at a voltage of 35 kV, with mass parameters set to *m*/*z* 50–1500. Data were evaluated utilizing DataAnalysis^TM^ software version 3.4 and Biotools software version 3.2 (Bruker Daltonics, Bremen, Germany). Peptide identification was evaluated using the PEAKS scoring system (Waterloo, ON, Canada), synthesizing only peptides with confidence scores exceeding 90%, hence providing dependable sequence assignments for the assessment of DPP-IV and ACE inhibitory activities.

#### 2.2.4. Peptide Synthesis and In Silico Gastrointestinal Digestion

Twenty-six selected peptides with significant inhibitory efficacy were made via solid-phase peptide synthesis (SPPS) (GL Biochem, Shanghai, Ltd., Shanghai, China). The synthesized peptides revealed a purity over 98%, and the manufacturer confirmed the molecular mass of the synthetic peptides using LC-MS/MS analysis. The peptides were solubilized in deionized water, and the resultant soluble peptides were evaluated for DPP-IV inhibitory activity at a concentration of 2.5 mM and ACE inhibitory activity at 0.2 mM (final reaction concentration). Peptides exhibiting significant inhibitory potential (>40% inhibition of both ACE and DPP-IV activities) were subsequently chosen for IC_50_ analysis.

Gastrointestinal digestion was simulated in silico using Pepsin, Trypsin, and Chymotrypsin with the Comprehensive Database of Food-Derived Bioactive Peptides for Peptidomics Research (DFBP) tool (http://www.cqudfbp.net/). The bioactive properties of both parent peptides and their fragments were investigated using the BIOPEP database. Novel peptide fragments (sequences not previously reported) were assessed for DPP-IV inhibitory activity at a concentration of 2.5 mM and ACE inhibitory activity at 0.2 mM (final reaction concentration). Peptides showing high potential (>40% inhibition of both ACE and DPP-IV activities) were subjected to IC_50_ determination.

**Figure 2:**
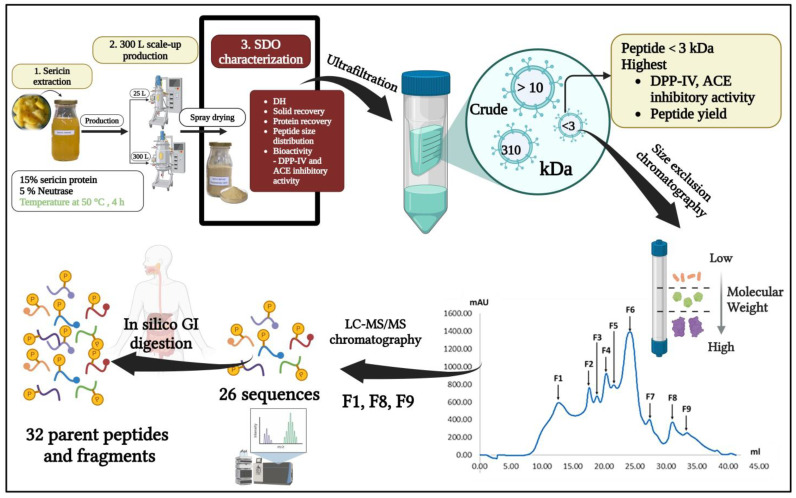
Schematic overview of SDO production and bioactive peptide identification workflow.

Figure 2 shows the detailed procedure that refines and characterizes crude SDOs in a systematic manner. Five steps were involved in the process: (1) the extraction of sericin and production scale-up to 300 L, (2) the characterization of the product including hydrolysis degree, solid recovery, and enzyme inhibitory activities, (3) molecular weight separation via ultrafiltration (<3 kDa, 3–10 kDa, >10 kDa), (4) size-exclusion chromatographic fractionation to produce F1-F9 fractions, and (5) the identification of bioactive peptides in selected fractions (F1, F8, F9) through LC-MS/MS analysis (26 peptides) and in silico gastrointestinal digestion simulation, resulting in 32 characterized bioactive sequences.

### 2.3. Commercial Quality Validation and Specifications

#### 2.3.1. Safety Assessments

##### Trace Metals Analysis

The analysis of trace metal content, specifically (Arsenic (As), Cadmium (Cd), Lead (Pb), and Mercury (Hg) in SDO powders was conducted at Central Laboratory (Thailand) Co., Ltd., Khon Kaen branch (Khon Kaen, Thailand) following the in-house method based on EPA 3052.

##### Microbial Analysis

The microbial analysis of SDO powders was also conducted at Central Laboratory (Thailand) Co., Ltd., Khon Kaen branch (Khon Kaen, Thailand). The tests included Coliforms and *Escherichia coli* (according to FDA BAM, 2017, Chapter 4) [36], *Listeria monocytogenes* (according to ISO 11290-1: 2017 (E)) [37], *Salmonella* spp. (according to ISO 6579-1: 2017 (E)) [38], *Staphylococcus aureus* (according to FDA BAM *Online*, 2016 (Chapter 12)) [39], Total Plate Count (according to FDA BAM, 2001 (Chapter 3)) [40], and Yeasts and Molds (according to FDA BAM, 2001 (Chapter 18)) [41].

##### Pesticide Residue Analysis

The pesticide residue analysis in SDO powders was conducted at Central Laboratory (Thailand) Co., Ltd., Bangkok branch (Bangkok, Thailand). The method was an in-house method based on QuEChERS, Method EN 15662:2018 [42]. The analysis focused on detecting residues of the carbamate group (using LC-MS), the organochlorine group and organophosphate group (using GC/µECD), and the pyrethroid group (using GC/FPD).

##### Total Protein Content and Amino Acid Profile Analysis

Total protein content was determined using Kjeldahl’s method as detailed in the AOAC [30] procedure by multiplying the percentage of total organic nitrogen in SDOs with 6.25 [30]. The amino acid profile of SDOs was analyzed at Central Laboratory (Thailand) Co., Ltd., Songkhla branch (Songkhla, Thailand). The in-house method used for analysis was based on the AOAC [30].

#### 2.3.2. Shelf Life and Stability of SDO Powders

To monitor changes in SDO powders while storage at 4 °C, 25 °C, and 45 °C during a six-month period as previously described by Nishanthi et al. [43], the measurements, including moisture content and water activity (Aw), were evaluated using the AOAC [30] approach as outlined by Baur and Ensminger [30]. A sample of SDO powder (1–5 g) was measured and placed onto a pre-weighed aluminum dish. The specimen was then placed in a hot-air oven (Memmert, Model 600, Schwabach, Germany) and dried at 105 °C overnight until a stable weight was obtained. The final weight was documented to determine the moisture content of the sample using the following calculation. (AOAC, [30]). The moisture content was determined as follows: Moisture content (%) = [(Initial weight − Final weight)/Initial weight] times 100. Water activity measurement was conducted using an Aqua lab water activity measurement equipment according to the manufacture’s procedure (Aqua Lab series 3 TE, Decagon Devices, Inc., Pullman, WA, USA). IC_50_ values for the inhibitory activity for ACE and DPP-IV enzymes were investigated following the procedures outlined in Section 2.2 and Section 2.3, respectively.

#### 2.3.3. Bioactive Properties

##### ABTS Radical Scavenging Activity Analysis

The antioxidant activity was evaluated using a modified ABTS assay according to Laosam et al. [32]. Then, ABTS stock solution was prepared by combining 7.0 mM ABTS with 2.6 mM potassium persulfate in 50 mM phosphate buffer (pH 7.4) and incubated in darkness for 12–16 h before use. Subsequently, the analysis was initiated by combining 20 μL of SDOs samples with 1980 μL of ABTS working solution (stock solution diluted with phosphate buffer to achieve an absorbance of 0.740 ± 0.020 at 734 nm). After 5 min of incubation in the dark, the absorbance was measured at 734 nm. The results were expressed as IC_50_ values (mg/mL of SDOs) compared against Trolox as a standard (Trolox: 0.27 mg/mL). With the formula below, the radical scavenging activity was calculated:

Scavenging activity (%) = [(A_control_ − A_sample_)/A_control_] × 100

where A_control_ and A_sample_ represent the absorbance of control and sample, respectively.

##### Ferric Reducing Antioxidant Power (FRAP) Analysis

A modified FRAP experiment based on Boonkong et al. [31] was used to analyze the reducing power. Fresh FRAP reagent was prepared by mixing 300 mM acetate buffer (pH 3.6), 10 mM TPTZ in 40 mM HCl, and 20 mM FeCl_3_•6H_2_O in a 10:1:1 ratio (*v*/*v*). For the analysis, 100 μL of the sample and 1 mL of FRAP reagent were combined, and the mixture was then allowed to sit at room temperature for 15 min. Absorbance was quantified at 593 nm, with results shown as IC_50_ values (mg/mL of SDOs). Trolox (1 mg/mL) served as a reference standard denoting 100% antioxidant activity.

##### Metal Chelating Activity Analysis

Assessment of metal chelating capacity was achieved using a modified method of Laosam et al. [44]. The procedure involved combining 50 μL of SDOs solution at a concentration of 50 mg/mL that was subsequently diluted into serial concentration solutions at 1, 5, 10, 20, 30, and 40 mg/mL, respectively, for IC_50_ determination with 1200 μL of deionized water and 25 μL of 2 mM FeCl_2_. Following a 3 min dark incubation, 50 μL of 5 mM ferrozine was introduced and incubated for 20 min at ambient temperature in the absence of light. Absorbance was quantified at 562 nm. Results were presented as IC_50_ values (mg/mL of SDOs) and compared to EDTA as a reference (0.31 mg/mL). The chelating activity was determined using the following formula:

Metal chelating activity (%) = [(A_control_ − A_sample_)/A_control_] × 100

### 2.4. Data Analysis

All experiments were conducted in triplicate. Data from the characterization of SDOs, as well as the shelf life and stability of SDO powders, were presented as the mean ± standard deviation (SD). The data were subjected to analysis of variance (ANOVA) using SPSS version 25 (SPSS Inc., Chicago, IL, USA) to determine significant differences among the means. Statistical significance was established at *p* < 0.05.

## 3. Results and Discussion

### 3.1. Production Scale-Up and Process Validation

#### 3.1.1. Effect of Scaling up SDO Production on Degree of Hydrolysis, Protein Recovery, and Bioactive Properties

This work demonstrates a significant increase in SDO production, which is distinguished by improved bioactive qualities attained through efficient upscaling, particularly evidenced by enhanced enzymatic efficiency and more uniform peptide generation due to superior control over critical parameters (temperature, pH, mixing) at larger scales. Expanding on our prior research [20], optimized production conditions, resulting in significant enhancements in yield and bioactivity at greater scales. The solid recovery and degree of hydrolysis (DH) increased gradually as the process was scaled up from laboratory scale to 25 L and 300 L capacities (Table 1). At the 300 L scale, we achieved 8.63% DH, 94.35% solid recovery, and 91.23% protein recovery—substantially higher than laboratory scale parameters of 5.02% DH, 85.23% solid recovery, and 85.36% protein recovery. The increased DH suggests more efficient peptide generation and potentially shorter peptide fragments, while the improved recovery rates indicate more consistent peptide production. These results indicate high potential economic advantages for commercial production.

The bioactivity most significantly increased with scale, peaking at 300 L capacity in terms of enzyme inhibition. DPP-IV inhibition enhanced from 44.64% at the laboratory scale to 49.83% at 300 L, whereas ACE inhibition rose from 62.19% to 65.83%. These improvements in biological activity provide functional evidence that the scale-up process maintains or enhances the quality and consistency of the bioactive peptides produced. Our work differs from other research in that it shows a notable increase in bioactivity with greater production scale. As Dullius et al. [45] observed in whey protein hydrolysis, industrial-scale processing enables superior control over critical parameters like temperature, pH, and mixing efficiency. Better control makes it easier for enzymes and substrates to interact, which maximizes the generation of peptides. Our findings expand upon recent work by Remme et al. [46], who reported similar bioactivity improvements when scaling up cod protein hydrolysate production from laboratory to pilot scale.

An essential accomplishment is the concurrent improvement in both DH and bioactivity. Generally, elevated DH is associated with diminished bioactivity resulting from excessive peptide degradation [47]. However, our optimized process parameters overcame this limitation, producing higher yields while maintaining and even improving enzyme inhibition properties. The process showed consistent improvements across all parameters from laboratory to 25 L to 300 L scales, demonstrating robust scalability [48]. This successful scale-up to 300 L has significant implications for commercial SDO production, suggesting potential for industrial-scale manufacturing while preserving SDOs’ bioactive properties.

These findings contribute valuable insights to the field of bioactive peptide production, demonstrating that careful optimization of processing parameters can enable successful industrial-scale production without compromising bioactivity. The enhanced enzyme inhibition properties at larger scales suggest potential advantages for commercial manufacturing of SDOs targeting metabolic disorders.

#### 3.1.2. Concentration Through Ultrafiltration (UF)-Based Separation of SDOs Produced at a 300 L Capacity

The 300 L scale SDOs were separated using ultrafiltration, which provided crucial information about the bioactivity and size distribution of peptides. Table 2 showed the fractionation of separated SDO peptides into three distinct molecular weight ranges: UF1 (>10 kDa), UF2 (3–10 kDa), and UF3 (<3 kDa). Notably, the UF3 fraction demonstrated superior enzyme inhibition, with 66.77% DPP-IV and 76.89% ACE inhibition—significantly higher than the crude solution’s 49.83% and 65.83%, respectively. Combined SDO peptides from UF2 and UF3 fractions yielded more than 97%, with UF3 contributing 55.47% and UF2 contributing 41.95%.

Lower molecular weight SDO peptides’ unique molecular interactions with enzyme binding sites may be responsible for their increased bioactivity. DPP-IV and ACE are proteolytic enzymes characterized by unique substrate affinities and binding mechanisms. DPP-IV specifically cleaves dipeptides from the N-terminus of peptides containing proline or alanine in the second position [49,50]. The UF3 peptides (<3 kDa) effectively inhibited DPP-IV by mimicking this structure while resisting cleavage. Similar to this, ACE prefers substrates that include hydrophobic amino acid residues at the three C-terminal locations. Small peptides that fit these requirements can bind to the enzyme’s active site competitively and function as strong inhibitors [51,52]. The enhanced inhibition noted in the UF3 fraction corresponds with the findings of Webster et al. [53], who illustrated that peptides < 3 kDa induced negligible steric hindrance, facilitating an optimal fit within enzyme binding sites.

Most significantly, the high combined yield (>97%) and potent bioactivity of SDO UF2 + UF3 fractions suggest that extensive fractionation may be unnecessary for commercial production. The presence of bioactive peptides in SDOs with an appropriate molecular weight in the total SDO hydrolysate already suggests the possibility of more efficient production processes. The potential to reduce processing stages and related expenses without sacrificing therapeutic efficacy makes this finding significant for industrial scale-up. The results align with recent trends in bioactive peptide production, where process optimization focuses on maximizing the yield of targeted molecular weight ranges rather than extensive purification [54].

### 3.2. Product Characterization and Bioactivity

#### 3.2.1. Purification and Identification of the Bioactive Peptides Present in SDOs

##### Size Exclusion Chromatography

SEC was used for purifying the bioactive peptides present in SDOs. The UF3 fraction (<3 kDa), which had stronger inhibitory effects on both DPP-IV and ACEs, was then separated on a Superdex peptide column, making nine different fractions. Significant relationships between the molecular weight of peptides, recovery yields, and their bioactivity properties were found using the fractionated process.

**Figure 3:**
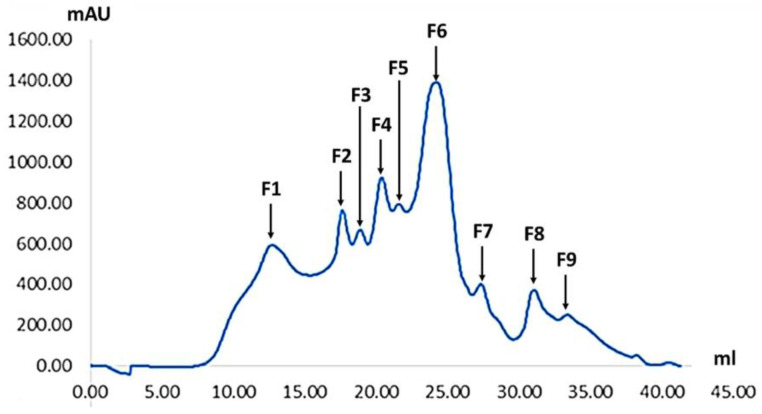
Size exclusion chromatogram showing the molecular weight distribution of SDO powders after spray drying at a concentration of 5 mg solid/mL.

Nine fractions (SF1-F9) with various retention durations and peak intensities were produced by the clear separation of peptides with varied molecular weights in our SEC fractionation profile (Figure 3). Notably, F6 showed the highest peak intensity around 1400 mAU, followed by prominent peaks for F3-F5, while later fractions (F7-F9) exhibited lower intensities. A particularly striking finding from Table 3 is the remarkable efficiency of ACE inhibition compared to DPP-IV inhibition. Although a lower concentration was used in the reaction mixture (1.0 mg Leu. eq./mL for ACE compared to 2.0 mg/mL for DPP-IV), multiple fractions exhibited enhanced ACE inhibitory activity. F1 exhibited an exceptional ACE inhibition (83.49 ± 0.77%) while requiring only half the sample concentration needed for DPP-IV inhibition (27.76 ± 0.57%). Similarly, F8 and F9 showed strong ACE inhibition (40.77% and 46.89%, respectively) at the lower concentration. This higher potency for ACE inhibition suggests these peptides may have structural features particularly favorable for ACE binding.

An interesting pattern emerges when evaluating peptide recovery alongside with bioactivity. Although the recovery rates of F6 and F7 were the greatest (37.24% and 19.41%, respectively), their profiles of enzyme inhibition were moderate. In contrast, F1, F8, and F9, despite lower recovery rates (3.11%, 1.55%, and 0.69%, respectively), demonstrated remarkable ACE inhibition efficiency. This inverse relationship between yield and specific activity aligns with findings by McCarthy et al. [55], who observed that highly bioactive peptides often represent a small fraction of total hydrolysates. Since these fractions had the most promising potential for therapeutic applications targeting ACE inhibition, we chose F1, F8, and F9 for additional sequencing analysis based on these results, especially the high ACE inhibition efficiency at lower concentrations.

##### Novel Peptide Identification and Simulated In Silico Gastrointestinal Digestion

**Figure 4:**
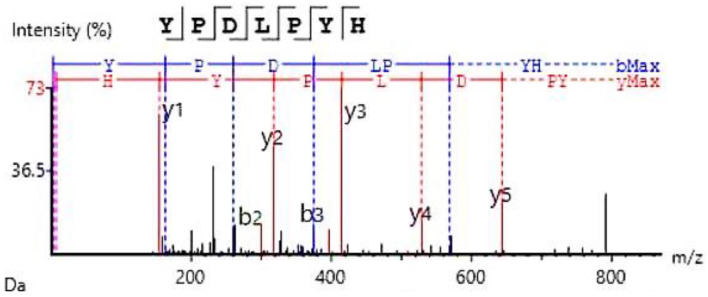
Mass spectrometry spectrum of SDO peptide fragmentation showing characteristic y- and b-ion series of YPDLPYH.

Several unique parent peptide sequences, spanning from 644.34 to 1135.44 Da, which constitute previously unreported bioactive compounds, were discovered during our analysis of SDOs from yellow silk cocoons. Through LC-MS/MS analysis, we identified 26 unique peptide sequences across fractions F1, F8, and F9 (Table 4). An example of the LC-MS/MS profile of the identified peptide YPDLPYH is shown in Figure 4. These identified peptides exhibited notable structural features and therapeutic potential. The molecular mass distribution is especially significant for maintaining stability during gastrointestinal transit while retaining bioactive properties [50].

Important patterns influencing the SDO peptides’ bioactivity were identified by analyzing the structure-activity relationship [48]. Many sequences contain specific structural motifs that enhance enzyme inhibition. For example, peptides containing C-terminal proline residues, such as VAEASLPR and LATWLPR, showed significant ACE inhibitory activity. Proline-containing peptides exhibit improved bioavailability and therapeutic efficacy due to their increased resistance to enzymatic degradation, which is why this structural feature is particularly significant [51].

A key advancement was the identification of peptides containing specific amino acid sequences associated with both ACE and DPP-IV inhibition. The peptides EFFDLPYH and NFDLPYH, both containing the PY motif, demonstrated enhanced DPP-IV inhibitory activity. The presence of hydrophobic amino acids at specific positions in these sequences also contributed to their ACE inhibitory properties [56,57]. The novel peptide QTALVELLQ (1013.58 Da) proved particularly versatile, exhibiting ACE, DPP-IV, and α-glucosidase inhibitory activities even after simulated gastrointestinal digestion.

The preservation of SDOs’ bioactivity after digestion observed in our in silico studies aligns remarkably well with our previous in vivo research [21]. Tocharus and Sutheerawattananonda [21] demonstrated significant hypoglycemic effects in STZ-induced diabetic rats, while Tocharus, Prum and Sutheerawattananonda [22] confirmed hypotensive effects in rodent studies. The findings confirm our in silico predictions and indicate that the digested fragments of SDOs retain their bioactive therapeutic properties in physiological conditions. Sangsawad, Katemala, Pao, Suwanangul, Jeencham and Sutheerawattananonda [20] previously showed that these peptides present in SDOs remain stable during both gastrointestinal and plasmin digestion, maintaining their dual DPP-IV and ACE inhibitory activities.

The simulation of gastrointestinal digestion demonstrated that these peptides produce di- and tripeptides that are efficiently absorbed via PepT1 transporters, whereas longer peptides (3–5 amino acids) employ different absorption mechanisms, including transcytosis and paracellular transport through tight junction proteins [58]. This diverse absorption profile enhances bioavailability, particularly for smaller peptides (<1000 Da), which exhibit superior absorption rates [59].

Consequently, throughout simulated gastrointestinal digestion, the peptides in SDOs maintain their bioactive therapeutic qualities, leading to the synthesis of absorbable di- and tripeptides. The stability and bioavailability of these SDO peptides under physiological settings are confirmed by the correlation between our in silico results and earlier in vivo investigations, making them attractive options for possible bioactive therapeutic applications.

#### 3.2.2. In Silico Gastrointestinal Digestion of SDOs and Bioactive Properties

For possible food applications, the bioactive qualities of synthetic peptides with amino acid sequences comparable to those of water-soluble SDO peptides and their gastrointestinal digestion fragments were analyzed (Table 5). The analysis focused on IC_50_ values for both parent sequences and their digestive fragments to assess their effectiveness as functional food ingredients. It is intriguing that a number of parent peptides maintained a high level of bioactivity, and their fragments exhibited inhibitory effects that were comparable or more potent, even after simulated gastrointestinal digestion. For instance, the parent peptide CEF (fragment of WCEFPR) showed remarkable ACE inhibition (91.67 ± 0.73%) compared to its parent sequence (75.43 ± 1.53%), suggesting a potential enhancement of SDOs bioactivity through the degradation of peptides during digestive processing.

The novel peptide sequences demonstrated remarkable dual inhibitory activities against both DPP-IV and ACEs. With a DPP-IV inhibition of 56.92 ± 1.47% (IC_50_ = 1.35 ± 0.04 mM) along with an ACE inhibition of 85.96 ± 1.66% (IC_50_ = 18.10 ± 0.05 µM), YPDLPYH (No. 24) exhibited exceptional dual inhibitory properties. The dual inhibitory activity of YPDLPYH compares favorably with several well-characterized peptide inhibitors. For DPP-IV inhibition, our peptide shows comparable potency to commercially successful peptides derived from milk proteins, such as β-lactoglobulin fragments with IC_50_ of 0.075–1.7 mM [60]. The ACE inhibitory activity is significant, positioned among powerful marine protein hydrolysates with an IC_50_ of 50–200 μM [61]. Although YPDLPYH exhibits lower efficacy compared to pharmaceutical DPP-IV inhibitors such as sitagliptin, which has an IC_50_ of 0.018 mM [62], it presents benefits related to its natural, organically sourced origin and dual activity. This outcome establishes YPDLPYH as one of the most promising naturally derived dual inhibitors, particularly due to its stability and potential for large-scale manufacturing.

SDOs’ structural characteristics that improve their functionality as food bioactive components include the stability-promoting C-terminal proline residues (WGWNPR, WMHVR) and the well-positioned aromatic amino acids (YPDLPYH, NFDLPYH) that promote improved enzyme inhibition. These characteristics contribute to both bioactivity and stability during food processing. This observation aligns with established mechanisms of peptide-enzyme interactions [63]. Acidic residues (Asp and Glu) may chelate zinc atoms necessary for enzyme function, whereas some amino acids (Tyr, Phe, Trp, Pro, Lys, Ile, Val, Leu, Arg) significantly affect enzyme binding [52,62,63]. This made the impact of amino acid composition in ACE inhibition especially noteworthy. The discovery of strong ACE inhibitors (CEF, YLYNAGAR, YPDLPYH) and DPP-IV inhibitors (YPDLPYH, NFDLPYH, EFYELAR, VAEASLPR) has been an important advancement. Interestingly, these previously unreported sequences possess ideal structural characteristics, including charged amino acids and proline residues positioned strategically, which support their dual inhibitory properties.

The identified peptides in SDOs demonstrate complementary bioactivities and stability profiles suitable for functional food applications. Parent peptides like YPDLPYH (56.92% DPP-IV inhibition) and their digestive fragments maintain activity through gastrointestinal transit, which supports their potential as functional food ingredients. The simultaneous presence of both parent peptides and their fragments appears to create a powerful therapeutic effect through complementary mechanisms. For DPP-IV inhibition, we identified several potent sequences working in concert: YPDLPYH (56.92%, IC_50_ = 1.35 mM), EFYELAR (56.23%, IC_50_ = 1.43 mM), and NFDLPYH (55.54%, IC_50_ = 2.38 mM), along with their bioactive fragments such as YPDL (48.28%, IC_50_ = 2.39 mM). Similarly, for ACE inhibition, multiple sequences demonstrated significant activity: YLYNAGAR (89.20%), YPDLPYH (85.96%, IC_50_ = 18.10 µM), and WCEFPR (75.43%, IC_50_ = 21.18 µM), with fragments like CEF showing remarkable potency (91.67%, IC_50_ = 14.86 µM). The combined action of these bioactive sequences in SDOs most likely produced the strong bioactive therapeutic results seen in our earlier in vivo studies on the hypoglycemic effects in STZ-induced diabetic rats [21] and hypotensive effects in rodent models [22]. This multi-peptide mechanism explains these results. The gastrointestinal digesting stability of these peptides, as verified by Sangsawad, Katemala, Pao, Suwanangul, Jeencham and Sutheerawattananonda [20], guarantees that both the parent peptides and their fragments retain their bioactive properties under physiological conditions. This provides a sustained and multi-targeted therapeutic effect, possibly through oral administration.

### 3.3. Commercial Quality Specifications

#### 3.3.1. Physicochemical and Safety Assessments of SDOs

##### Trace Metals and Microbial Contamination

The safety analysis for trace metals and microbial contamination is shown in Table 6. Trace metal content in SDO powders fell within acceptable regulatory limits; thus, they are considered safe from trace metal contamination. Cadmium (Cd) and mercury (Hg) were not found in SDO powders, whereas arsenic (As) and lead (Pb) were detected at low levels. The findings met the criteria for food containing contaminants in Thailand, as outlined in the Ministry of Public Health Notification (No. 414) (2020), issued under the Food Act (1979). The regulation specifies that in dietary supplement products, the maximum allowable levels are 0.3 ppm for Cd, 2 ppm for Hg, 2 ppm for As, and 1 ppm for Pb.

The SDO powder sample showed no contamination of *Listeria monocytogenes* and *Salmonella* spp. Microbial analysis showed that levels of Coliforms., *Escherichia coli*, *Staphylococcus aureus*, total plate count, and yeasts and molds were found to be within the standard (Table 6). This is in line with the Ministry of Public Health Notification Thailand standards for pathogenic microorganisms in food. For concentrated or dry beverage products, it specifies that *Salmonella* spp. and *Listeria monocytogenes* must not be detected in a 25 g sample.

##### Pesticide Residues

Four primary pesticide categories were examined in the analysis of pesticide residues in SDO powders (Table 7): carbamate, pyrethroid, organochlorine, and organophosphate. All analyzed pesticides were below detectable thresholds, adhering to food safety standards for functional food components. This thorough test encompassed frequently monitored agricultural pesticides, including Carbaryl, Deltamethrin, DDT, and Chlorpyrifos, thereby affirming the safety of SDOs for food applications. The pyrethroid class of insecticides, including Permethrin and Cyfluthrin, is extensively utilized for agricultural pest management and is categorized with newer compounds such as Fenvalerate [64]. These compounds are recognized for their efficacy in targeting a wide range of insects. Nonetheless, their presence in food products prompts concerns regarding possible human health hazards if present in significant amounts [65]. Since there are no numerical values, it is possible that the study found very low amounts or none at all, ensuring adherence to food safety regulations. The Organophosphate group, recognized for its neurotoxic properties [66], includes strictly regulated compounds, including Fenitrothion, Parathion, and Chlorpyrifos, which are regularly inspected in food safety assessments due to their toxicological effects [67]. Their detection, if higher than the maximum residue limit, could have considerable impacts on public health, especially for enzyme inhibition studies involving assessments of ACE and DPP-IV inhibitory activity. These enzymes are crucial in managing hypertension and diabetes [6,7,8], respectively. Thus, any contamination with organophosphates could affect the bioactivity and safety profile of the SDO powders. Therefore, ensuring that pesticide residues are within safe limits is critical for maintaining both the therapeutic potential and consumer safety of SDOs, sericin-derived products from yellow silk cocoons.

##### Amino Acids Composition

SDO powder’s amino acid composition on a dry basis (Table 8) suggests a nutritional profile sufficient for its application as a functional food ingredient similar to the previous research by Puangphet et al. [68]. The 19 amino acids included key ones such as serine (20.50 g/100 g), aspartic acid (16.89 g/100 g), glycine (5.22 g/100 g), and glutamic acid (4.90 g/100 g). In addition to being necessary for many physiological functions, these amino acids probably play a role in the health advantages of SDOs from yellow silk cocoons, including their ACE and DPP-IV inhibitory effects.

The amino acid composition explains the functional attributes of SDOs as a dietary component. The high serine amount (20.50 g/100 g) facilitates ACE inhibition via enzyme binding, whereas proline and glycine strengthen DPP-IV inhibitory action [69]. Essential amino acids like leucine (2.40 g/100 g) and lysine (5.01 g/100 g) further enhance the nutritional value of SDOs as a functional food ingredient [69]. Glutamic acid, a negatively charged amino acid, may facilitate ACE inhibition via electrostatic interactions, potentially obstructing the conversion of angiotensin I to angiotensin II, a mechanism associated with blood pressure management [70].

Proline, glycine, and phenylalanine are amino acids that may be essential for DPP-IV inhibition. DPP-IV recognizes and cleaves peptides with proline, indicating that proline may serve as a potential competitive inhibitor [71]. The incorporation of arginine and lysine, both cationic amino acids, may improve interactions with the enzyme, resulting in the reduction in its activity [72,73]. These interactions have the potential to prolong the positive effects of incretins, including GLP-1, on insulin production and glucose homeostasis by slowing down their breakdown [74]. This amino acid profile illustrates the potential of SDOs from yellow silk cocoons as a multifunctional food ingredient, integrating nutritional value with bioactive capabilities for applications in metabolic health.

#### 3.3.2. Stability Studies Supporting Shelf-Life Claims

Over a six-month period, the storage stability of SDOs was assessed at temperatures of 4, 25, and 45 °C, which are considered essential to applications in the food industry. Key food quality parameters including moisture content, water activity (A_W_), and retention of bioactive properties were monitored to establish shelf-life guidelines for food applications. These temperatures, which include ambient circumstances (25 °C), accelerated stability testing (45 °C), and chilled storage (4 °C), were selected to represent typical situations in homes, warehouses, logistics, and distribution. This analysis is necessary for assessing the shelf life and long-term quality of food products, as moisture content and aw greatly affect preservation, microbiological safety, and the physicochemical properties of powdered products.

At 4 °C, the initial moisture content of the SDOs was 2.64 ± 0.03%, and the A_W_ value was 0.409 ± 0.01. The moisture content fluctuated somewhat throughout time, increasing by a small amount to 2.67 ± 0.02% at the 3-month mark. Nevertheless, by the six-month mark, the moisture content decreased to 2.34 ± 0.15%, and the A_W_ value dropped to 0.382 ± 0.02, suggesting a decline in water retention. This drop likely indicates a progressive dehydration process at low temperatures, which may improve the powder’s stability [75]. Similar patterns were seen at 25 °C, although the moisture content gradually dropped in comparison to the 4 °C temperature. The moisture content decreased from 2.64 ± 0.03% at first to 2.24 ± 0.12% by the third month, maintaining stability at 2.24 ± 0.14% after six months. The A_W_ increased slightly to 0.420 ± 0.01 in three months before declining to 0.370 ± 0.02 in six months, indicating a balanced interaction between water migration and evaporation at room temperature [76]. Finally, at 45 °C, a notable reduction in both moisture content and A_W_ were observed more quickly compared to the lower temperatures. Beginning with the same starting moisture content and the A_W_ value of 2.64 ± 0.03% and 0.409 ± 0.01, respectively, the moisture content decreased to 2.23 ± 0.27% at three months and 2.13 ± 0.15% at six months, while A_W_ had a comparable trend, decreasing to 0.373 ± 0.01 by the six-month mark. These results imply that increased storage temperatures accelerate moisture loss [77], which could influence changes in the powder’s physical properties.

Table 9 presents the ACE and DPP-IV inhibitory activities, illustrating the stability of SDO powders throughout a six-month storage period at various temperatures. The findings show the IC_50_ values for ACE and DPP-IV inhibition throughout a 6-month period at three distinct storage temperatures (4 °C, 25 °C, and 45 °C). Interestingly, while both enzymes show some fluctuation in inhibition levels, they exhibited distinct patterns of change over time and under different temperature conditions. Regardless of temperature, DPP-IV inhibition showed a general tendency of rising IC_50_ values with increasing storage time. This indicates that the SDO powder’s capacity to inhibit DPP-IV diminished slightly with the longer storage time. The effect was more significant at elevated temperatures, with the 45 °C storage condition exhibiting the highest IC_50_ value (10.89 ± 0.24) after 6 months of storage, suggesting that elevated temperatures may expedite the decline in DPP-IV inhibitory activity. It is important to note that the powder’s DPP-IV inhibitory qualities showed considerable stability, as evidenced by the fact that the IC_50_ fluctuated slightly even at high temperatures and extended storage times. The SDO powder, in contrast, exhibited a higher degree of stability in its ACE inhibitory effect, regardless of temperature and time fluctuations. Throughout the six-month period, the IC_50_ values for ACE inhibition exhibit minimal fluctuation, with the majority of values remaining around 28–29 μg/mL. The IC_50_ value (29.40 ± 0.83), even at the maximum temperature of 45 °C after 6 months, was only marginally higher than the starting value. This exceptional stability implies that the compounds in the SDO powder that inhibited ACE were more resilient to degradation or structural alterations brought on by temperature and storage time than those that inhibited DPP-IV. The consistent ACE inhibitory activity may provide a considerable benefit for the prolonged storage and effectiveness of the SDO powder as a prospective functional or nutritional supplement addressing ACE-related health issues.

#### 3.3.3. Future Applications in Food Systems

These results indicate that SDOs have considerable potential for commercial food applications due to their advantageous physicochemical characteristics. Their powdered form, distinguished by a low moisture content (2.64%) and water activity (below 0.6), together with their stability over a range of storage temperatures (4–45 °C), makes them suitable for many food systems. The increased protein content (91%) and maintained dual bioactive functions (DPP-IV and ACE inhibition) throughout simulated digestion establish SDOs as significant functional components, consistent with contemporary trends in bioactive peptide application [78]. These attributes facilitate the integration of SDOs across several food categories. In beverage applications, their excellent solubility renders them appropriate for functional beverages and protein shakes, as shown by successful implementations of bioactive peptides in liquid forms, as reviewed by Saubenova et al. [79]. Chai et al. [80] emphasized the improved stability and bioavailability of peptides in fermented dairy products. The powdered form also enables integration into cakes, breads, baked goods, sausages, and patties [81]. The combined role of SDOs in delivering both hypoglycemic and antihypertensive effects constitutes a notable benefit in the advancement of functional foods. This outcome corresponds with the increasing market demand for multi-functional components [79,82,83]. Their stability under diverse processing circumstances facilitates use in ready-to-drink supplements and fortified nutritional products, following successful commercialization trends in bioactive peptides [58,79,84].

**Figure 5:**
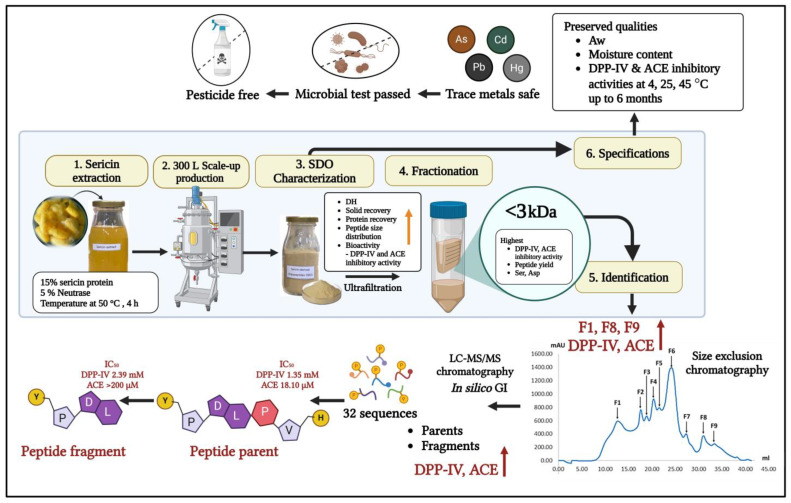
Schematic overview of SDO production process and characterization.

In summary, our systematic approach to SDO production and characterization (Figure 5) demonstrates the comprehensive development of a safe and stable bioactive product. Beginning with the first extraction, which contained 15% sericin from yellow silk cocoons and 5% Neutrase (E/S), the procedure was effectively expanded to 300 L while preserving quality. Through ultrafiltration and size exclusion chromatography, we isolated the most potent peptide fractions (F1, F8, F9) with molecular weights < 3 kDa, which showed optimal DPP-IV and ACE inhibitory activities. Through LC-MS/MS analysis, 32 novel peptide sequences and their fragments were identified, and structure-activity interactions were discovered, providing evidence for their bioactive therapeutic potential. Critically, our production procedure confirms product safety by conducting thorough testing for trace metals (As, Cd, Pb, Hg), microbiological contamination, and pesticide residues in accordance with food safety requirements while ensuring stability at 45 °C for a duration of up to 6 months.

Nonetheless, certain limitations of this study must be considered. Although our in silico digestion simulations offer significant insights, they may not completely mimic the complexity of the actual digestive system and its possible impacts on peptide bioavailability. Secondly, while we established stability at 45 °C for a duration of 6 months, it is imperative to conduct long-term stability experiments under multiple adverse storage circumstances for commercial applications and logistic conditions. Furthermore, although we have identified certain bioactive sequences, additional exploration of their unique binding mechanisms via molecular docking studies could provide enhanced understanding of their structure-activity correlations. These limitations provide possibilities for future research to improve our comprehension of SDOs’ bioactive therapeutic potential and refine their commercial development.

## 4. Conclusions

Through meticulous development from production to stability validation, this study successfully established SDOs from yellow silk cocoons as a novel functional food ingredient. The optimized 300 L scale production achieved superior performance metrics (8.63% degree of hydrolysis, 94.35% solid recovery) while enhancing bioactive properties. Additionally, 32 distinct peptide sequences were identified through advanced characterization, including YPDLPYH, which exhibited extraordinary stability and dual-enzyme inhibition (DPP-IV IC_50_ 1.35 mM; ACE IC_50_ 18.10 μM) during simulated digestion. Trace metals, pesticides, and bacterial contaminants were below the safety maximum residue limits and complied with all food-grade standards. Stability studies revealed that SDO powders maintained both physical and functional properties during six months of storage at temperatures of 45 °C, with water activity remaining below 0.6—an essential criterion for food applications. This study offers food manufacturers a confirmed approach for producing sustainable, bioactive components derived from sericin in yellow silk cocoons, an organic silk protein, suitable for various commercial applications, including functional beverages, nutritional supplements, and fortified food products targeting metabolic health enhancement. The demonstrated stability and functionality of SDOs under different processing conditions support their versatility as a functional food ingredient. Future work should focus on optimizing food matrix incorporation and developing specific product applications. By successfully transferring a novel bioactive component from laboratory discovery to commercial-scale production, this work supports sustainable manufacturing practices, the bio-circular green economy, and innovation in the food industry.

## Figures and Tables

**Table 1:** Enzymatic inhibition for DPP-IV and ACE, recovery yields, and hydrolysis degree of SDOs during the production scale-up.

Fractions	Inhibition (%)		Degree of Hydrolysis (%)	Solid Recovery (%)	Protein Recovery (%)
	DPP-IV	ACE			
(1) LAB scale	44.64 ± 3.21 ^b^	62.19 ± 1.40 ^b^	5.02 ± 0.88 ^b^	85.23 ± 0.33 ^c^	85.36 ± 2.86 ^b^
(2) Up-scale 25 L	47.32 ± 2.43 ^ab^	63.22 ± 1.43 ^b^	5.36 ± 1.02 ^b^	90.34 ± 0.21 ^b^	87.13 ± 1.24 ^b^
(3) Up-scale 300 L	49.83 ± 1.59 ^a^	65.83 ± 0.59 ^a^	8.63 ± 0.92 ^a^	94.35 ± 0.70 ^a^	91.23 ± 0.23 ^a^

The DPP-IV and ACE inhibitory activities were measured in the reaction mixture at final concentrations of 2.0 mg and 1.0 mg Leucine equivalents per mL (Leu. eq./mL), respectively. Significantly different values (*p* < 0.05) are represented by superscript letters (^a–c^) within the column.

**Table 2:** Bioactive properties and the peptide yield obtained from the UF-based separation of SDOs at a production scale of 300 L.

Fraction	Inhibition (%)		SDO Peptide Yield (%)
	DPP-IV	ACE	
Crude solution	49.83 ± 1.59 ^b^	65.83 ± 1.59 ^b^	-
UF1 (>10 kDa)	8.18 ± 5.93 ^d^	18.32 ± 2.34 ^d^	2.70 ± 1.12 ^c^
UF2 (3–10 kDa)	24.79 ± 6.65 ^c^	44.42 ± 4.52 ^c^	41.95 ± 0.02 ^b^
UF3 (< 3 kDa)	66.77 ± 4.79 ^a^	76.89 ± 2.64 ^a^	55.47 ± 5.57 ^a^

The DPP-IV and ACE inhibitory activities were measured in the reaction mixture at final SDOs concentrations of 2.0 mg and 1.0 mg Leu. eq./mL, respectively. Significantly different values (*p* < 0.05) are represented by superscript letters (^a–d^) within the column.

**Table 3:** DPP-IV and ACE inhibition and the peptide recovery of each fraction of SDOs with a molecular weight less than 3 kDa.

SDOs Samples	Inhibition (%)		Peptide Recovery (%)
	DPP-IV	ACE	
F1	27.76 ± 0.57 ^ab^	83.49 ± 0.77 ^a^	3.11 ± 0.03 ^de^
F2	24.14 ± 0.93 ^cd^	10.57 ± 0.45 ^de^	2.73 ± 0.00 ^e^
F3	23.19 ± 0.19 ^d^	2.90 ± 0.65 ^f^	4.08 ± 0.10 ^d^
F4	29.86 ± 1.08 ^a^	7.68 ± 1.30 ^e^	8.36 ± 0.28 ^c^
F5	25.67 ± 0.76 ^bcd^	7.89 ± 0.77 ^e^	7.32 ± 0.21 ^c^
F6	24.02 ± 0.95 ^cd^	11.26 ± 1.41 ^de^	37.24 ± 0.76 ^a^
F7	27.00 ± 2.10 ^abc^	13.76 ± 1.58 ^d^	19.41 ± 0.59 ^b^
F8	23.00 ± 0.00 ^d^	40.77 ± 1.61 ^c^	1.55 ± 0.00 ^f^
F9	18.81 ± 0.76 ^e^	46.89 ± 1.85 ^b^	0.69 ± 0.07 ^f^

The DPP-IV and ACE inhibitory activities were measured in the reaction mixture at final concentrations of 2.0 mg and 1.0 mg Leu. eq./mL, respectively. Significantly different values (*p* < 0.05) are represented by superscript letters (^a–f^) within the column.

**Table 4:** Identified SDO peptides derived from enzymatic hydrolysis of sericin from yellow silk cocoons.

Fractions	No.	Sequence	Mass (Da)	Peptide Fragment After Simulated In Silico Gastrointestinal Digestion *	Previous Reported Bioactivity of the Fragments (BIOPEP-UWM Database) *
F1	1	YLYESPR	926.45	Y-L-YESPR ^PT^	-
				Y-L-Y-ESPR ^PC^	-
	2	QTALVELLQ	1013.58	QTA-L-VE-L-LQ ^PT, PC^	(VE, LQ) ACE, DPP-IV, Alpha-glucosidase inhibitor
	3	VAEASLPR	841.47	VAEASL-PR ^PT, PC^	(PR) ACE, DPP-III inhibitor
	4	EFLLLHA	841.47	EFL-L-L-HA ^PT^	(HA) DPP-IV inhibitor
				EF-L-L-L-HA ^PC^	(EF, HA) ACE, Renin, DPP-IV inhibitor
	5	LATWLPR	855.50	LATWL-PR ^PT^	(PR) ACE, DPP-III inhibitor
				LATW-L-PR ^PC^	(PR) ACE, DPP-III inhibitor
	6	EFYELAR	926.45	EFYE-L-AR ^PT^	(AR) ACE inhibitor
				EF-Y-E-L-AR ^PC^	(EF, AR) ACE, Renin inhibitor
	7	AEFVEVTQ	921.44	AEF-VEVTQ ^PT, PC^	
F8	8	WWDSDNQ	949.36	W-W-DSDNQ ^PC^	-
	9	LFDQPYF	928.43	LF-DQPY ^PC^	(LF) ACE inhibitor
	10	LGNLYDY	856.40	LGN-L-YDY ^PT^	(YDY) Antioxidative
				LGN-L-Y-DY ^PC^	(DY) ACE inhibitor, Regulating
	11	YLYNAGAR	926.46	Y-LY-NAGAR ^PC^	(LY) ACE, Renin inhibitor, Antioxidative
	12	MEDLFLFH	1050.48	MED-L-F-L-FH ^PT^	-
				MED-L-F-L-F-H ^PC^	-
	13	EFFDLPYH	1066.48	EFF-DL-PYH ^PT^	-
				EF-F-DL-PY-H ^PC^	(EF, PY) ACE, Renin, DPP-IV inhibitor, Anti-inflammatory
	14	YAHVR	644.34	Y-AHVR ^PC^	-
	15	DDKVFH	759.36	DDK-V-FH ^PT^	-
				DDKV-F-H ^PC^	-
	16	LFDLMEH	903.42	LFD-L-MEH ^PT^	-
				LF-D-L-MEH ^PC^	(LF) ACE inhibitor
	17	WLSPAYF	882.43	W-LSPAY-F ^PC^	-
F9	18	WWWDSDNQ	1135.44	W-W-W-DSDNQ ^PC^	-
	19	WGWNPR	814.39	W-GM-NPR ^PC^	(GM) ACE, DPP-IV inhibitor
	20	WMHVR	727.36	MW-HVR ^PC^	(MW) ACE, DPP-IV inhibitor
	21	NFDLPYH	904.41	NFDL-PYH ^PT^	-
				NF-DL-PY-H ^PC^	(NF, PY) ACE, DPP-IV inhibitor, Anti-inflammatory
	22	WCEFPR	836.36	WCEF-PR ^PT^	(PR) ACE, DPP-III inhibitor
				W-CEF-PR ^PC^	(PR) ACE, DPP-III inhibitor
	23	ENSQQLH	854.39	ENSQQ-LH ^PT, PC^	(LH) DPP-IV inhibitor, Antioxidative
	24	YPDLPYH	903.41	YPDL-PYH ^PT^	-
				YPDL-PY-H ^PC^	(PY) DPP-IV inhibitor, Anti-inflammatory
	25	WSVNPDQ	844.37	W-SVNPDQ ^PC^	-
	26	WWMVH	757.34	W-WMVH ^PC^	-

* Data from BIOPEP-UWM database: http://www.uwm.edu.pl/biochemia/index.php/pl/biopep (accessed on 27 May 2024) ^PT^ Peptides are digested by Pepsin followed by Trypsin. ^PC^ Peptides are digested by Pepsin followed by Chymotrypsin. (-) means no data.

**Table 5:** The bioactive properties and IC_50_ value of the synthetic peptides are similar to those found in SDOs.

No	Peptide Parent	Peptide Fragment *	Inhibition (%)		Inhibition (IC_50_ Value)	
			DPP-IV ^(A)^	ACE ^(B)^	DPP-IV (mM)	ACE (µM)
2	QTALVELLQ		22.20 ± 0.26 ^n^	ND	-	-
		QTA	10.54 ± 1.90 ^o^	16.48 ± 1.26 ^kl^	-	-
3	VAEASLPR		55.20 ± 0.69 ^a^	3.89 ± 0.61 ^op^	1.92 ± 0.06 ^f^	-
4	EFLLLHA		40.46 ± 1.74 ^efgh^	58.32 ± 0.92 ^f^	3.57 ± 0.13 ^a^	138.35 ± 1.04 ^c^
		EFL	38.00 ± 1.38 ^fghi^	ND	-	-
5	LATWLPR		47.44 ± 1.73 ^bc^	36.15 ± 0.54 ^h^	2.03 ± 0.15 ^f^	-
6	EFYELAR		56.23 ± 1.55 ^a^	33.96 ± 2.33 ^hi^	1.43 ± 0.03 ^g^	-
		EFYE	36.03 ± 0.51 ^ghij^	ND	-	-
7		AEF	21.93 ± 0.35 ^n^	6.04 ± 1.41 ^o^	-	-
9	LFDQPYF		45.00 ± 2.04 ^bcde^	42.40 ± 2.64 ^g^	3.17 ± 0.17 ^b^	-
10	LGNLYDY		42.88 ± 0.09 ^cdef^	58.61 ± 2.61 ^f^	2.94 ± 0.09 ^c^	162.08 ± 1.19 ^a^
		LGN	35.84 ± 0.09 ^hij^	44.11 ± 0.96 ^g^	-	-
11	YLYNAGAR		37.02 ± 0.95 ^ghij^	89.20 ± 1.07 ^ab^	-	77.28 ± 0.05 ^e^
12		MED	38.87 ± 0.64 ^fghi^	ND	-	-
		FH	27.98 ± 0.69 ^lm^	31.29 ± 3.45 ^i^	-	-
13	EFFDLPYH		45.72 ± 1.86 ^bcd^	63.39 ± 1.60 ^e^	2.76 ± 0.03 ^cd^	158.26 ± 2.20 ^b^
		EFF	30.57 ± 1.97 ^kl^	ND	-	-
		DL	24.12 ± 0.70 ^mn^	12.93 ± 0.67 ^lmn^	-	-
15		DDK	37.22 ± 0.43 ^ghij^	ND	-	-
16	LFDLMEH		37.28 ± 0.69 ^ghij^	ND	-	-
		LFD	33.85 ± 1.27 ^ijk^	ND	-	-
		MEH	37.20 ± 3.18 ^ghij^	11.20 ± 0.04 ^n^	-	-
19	WGWNPR		37.45 ± 1.55 ^ghij^	23.69 ± 2.90 ^j^	-	-
		NPR	36.70 ± 0.09 ^ghij^	17.84 ± 0.68 ^k^	-	-
20	WMHVR		33.06 ± 2.67 ^jk^	61.29 ± 0.88 ^ef^	-	117.78 ± 1.82 ^d^
21	NFDLPYH		55.54 ± 0.34 ^a^	69.53 ± 0.03 ^d^	2.38 ± 0.03 ^e^	77.22 ± 2.70 ^e^
		PYH	29.97 ± 0.60 ^kl^	15.69 ± 1.82 ^klm^	-	-
22	WCEFPR		41.15 ± 0.43 ^defg^	75.43 ± 1.53 ^c^	2.82 ± 0.22 ^c^	21.18 ± 1.86 ^f^
		CEF	23.49 ± 0.14 ^mn^	91.67 ± 0.73 ^a^	ND.	14.86 ± 0.10 ^g^
23	ENSQQLH		40.44 ± 4.54 ^efgh^	ND	2.54 ± 0.10 ^de^	-
24	YPDLPYH		56.92 ± 1.47 ^a^	85.96 ± 1.66 ^b^	1.35 ± 0.04 ^g^	18.10 ± 0.05 ^f^
		YPDL	48.28 ± 0.59 ^b^	11.77 ± 2.26 ^mn^	2.39 ± 0.06 ^e^	-

Notes: (-) means no data, and ND means not determined. * means the peptide fragments were released after simulated in silico GI digestion. Data from BIOPEP-UWM database: http://www.uwm.edu.pl/biochemia/index.php/pl/biopep (accessed on 27 May 2024). ^(A)^ Assay at a concentration of 2.5 mM and ^(B)^ at a concentration of 0.2 mM (final reaction concentration). Significantly different values (*p* < 0.05) are represented by superscript letters (^a–p^) within the column.

**Table 6:** Trace metal content and microorganisms in SDO powders.

Trace Metals (ppm)		Microbiological Test		
Arsenic (As)	0.105	Coliforms	<3.0	MPN/g
Cadmium (Cd)	-	*Escherichia coli*	<3.0	MPN/g
Lead (Pb)	0.299	*Listeria monocytogenes*	-	per 25 g
Mercury (Hg)	-	*Salmonella* spp.	-	per 25 g
		*Staphylococcus aureus*	<10	CFU/g
		Total Plate Count	3.0 × 10^3^	CFU/g
		Yeasts and Molds	<10	CFU/g

Note: (-) nondetectable, and CFU means a colony-forming unit.

**Table 7:** Content of pesticide residues in SDO powders.

Pesticide Residues (ppm)					
Carbamate group		Pyrethroid group		Organophosphate group	
Carbaryl	-	Deltamethrin	-	Fenitrothion	-
Isoprocarb	-	Bifenthrin	-	Parathion	-
Fenobucarb	-	Permethrin	-	Prothiofos	-
Promecarb	-	lambda-Cyhalothrin	-	Methidathion	-
Carbofuran	-	Cypermethrin	-	Profenofos	-
Methiocarb	-	Cyfluthrin	-	Ethion	-
Methomyl	-	Fenvalerate	-	Triazophos	-
Aldicarb		Organophosphate group		EPN	-
Oxamyl	-	Dichlorvos (DDVP)	-	Phosalone	-
Metolcarb	-	Methamidophos	-	Azinphos-ethyl	-
Organochlorine group		Mevinphos	-		
BHC (HCH)	-	Omethoate	-		
Heptachlor&Heptachlor-epoxide	-	Diazinon	-		
Aldrin (HHDN) and Dieldrin (HEOD)	-	Dicrotophos	-		
Dicofol	-	Pirimiphos-methyl	-		
DDT	-	Chlorpyrifos	-		
Chlordane	-	Parathion-methyl	-		
Endosulfan	-	Pirimiphos	-		
Endrin	-	Malathion	-		

Note (-) nondetectable.

**Table 8:** Amino acids profile of SDO powder * (g/100 g dry weight basis).

Amino Acids	Content	Amino Acids	Content
Alanine	2.30 ^j^	Methionine	0.41 ^o^
Arginine	3.72 ^f^	Phenylalanine	0.74 ^n^
Aspartic acid	16.89 ^b^	Proline	0.82 ^m^
Cystine	0.23 ^p^	Serine	20.50 ^a^
Glutamic acid	4.90 ^e^	Threonine	5.20 ^c^
Glycine	5.22 ^c^	Tryptophan	-
Histidine	1.15 ^k^	Tyrosine	3.29 ^g^
Hydroxylysine	-	Valine	2.86 ^h^
Hydroxyproline	-	Asparagine	-
Isoleucine	0.71 ^n^	Cysteine	0.98 ^l^
Leucine	2.40 ^i^	Glutamine	2.30 ^i^
Lysine	5.01 ^d^	Total amino acid	79.63

* 2.64% moisture content with 91% total protein. Note (-) nondetectable. ^a–p^ means significantly different at *p* < 0.05.

**Table 9:** Stability of SDO powders during 6 months of storage.

Storage Condition		A_W_	Moisture Content (%)	IC_50_	
Temperature (°C)	Time (Month)			DPP-IV (mg/mL)	ACE (µg/mL)
4	0	0.409 ± 0.01 ^a^	2.64 ± 0.03 ^a^	7.76 ± 0.30 ^b^	28.44 ± 0.39 ^a^
	3	0.409 ± 0.02 ^a^	2.67 ± 0.02 ^a^	7.93 ± 0.17 ^b^	28.36 ± 0.10 ^a^
	6	0.382 ± 0.02 ^b^	2.34 ± 0.15 ^b^	9.76 ± 0.52 ^a^	28.63 ± 0.11 ^a^
25	0	0.409 ± 0.01 ^a^	2.64 ± 0.03 ^a^	7.76 ± 0.30 ^b^	28.44 ± 0.39 ^a^
	3	0.420 ± 0.01 ^a^	2.24 ± 0.12 ^b^	8.13 ± 0.14 ^b^	28.98 ± 0.22 ^a^
	6	0.370 ± 0.02 ^b^	2.24 ± 0.14 ^b^	9.86 ± 0.53 ^a^	28.62 ± 0.17 ^a^
45	0	0.409 ± 0.01 ^a^	2.64 ± 0.03 ^a^	7.76 ± 0.30 ^b^	28.44 ± 0.39 ^b^
	3	0.419 ± 0.01 ^a^	2.23 ± 0.27 ^b^	8.16 ± 0.18 ^b^	28.62 ± 0.19 ^b^
	6	0.373 ± 0.01 ^b^	2.13 ± 0.15 ^b^	10.89 ± 0.24 ^a^	29.40 ± 0.83 ^a^

^a,b^ Different letters in each column indicate statistically significant differences (*p* < 0.05).

## Data Availability

The original contributions presented in this study are included in the article. Further inquiries can be directed to the corresponding authors.
